# Explaining the Comprehension–Production Vocabulary Gap Through Neural Networks and Cross‐Syndrome Evidence: Insights From Williams Syndrome

**DOI:** 10.1111/desc.70115

**Published:** 2026-01-08

**Authors:** Dean D'Souza, Hana D'Souza, Julien Mayor, Ángel Eugenio Tovar

**Affiliations:** ^1^ Centre for Human Developmental Science School of Psychology Cardiff University Cardiff UK; ^2^ Department of Psychology University of Oslo Oslo Norway; ^3^ Facultad de Psicología Universidad Nacional Autónoma de México Mexico City México; ^4^ Centro de Ciencias de la Complejidad Universidad Nacional Autónoma de México Mexico City México

**Keywords:** categorisation, comprehension–production vocabulary gap, computational modelling, language development, self‐organising maps, Williams syndrome

## Abstract

The comprehension–production vocabulary gap is a well‐documented hallmark of language development; however, anecdotal evidence suggests that this asymmetry may be reduced in children with Williams syndrome (WS). Here, we use empirical data to characterise the comprehension–production gap and computational modelling to investigate potential mechanisms underlying this distinctive linguistic profile, focusing on children aged 7 months to 6 years. Using parental reports (Communicative Development Inventories), we measured the receptive and expressive vocabularies of children with WS (*n* = 67) and compared them to typically developing children (*n* = 1210) and cross‐syndrome groups with Down syndrome (*n* = 27), and fragile X syndrome (*n* = 15). Results confirm that children with WS show a unique trajectory: alongside general delay, they exhibit a significantly reduced comprehension–production asymmetry not observed in other groups. To elucidate the potential origins of this phenomenon, we implemented a biologically inspired neural network—self‐organising map (SOM)—to model early word learning and evaluate visual and auditory map representations. Our findings reveal that WS‐like vocabulary patterns can emerge from selective difficulties in visual processing, leading to exemplar‐based rather than prototype‐based object representations. The model suggests that these visual processing challenges, consistent with known visuospatial difficulties in WS, may contribute to the atypical comprehension–production relationship, while broader processing constraints may underlie general delays. This study provides a mechanistic account of vocabulary development in WS, highlighting the role of visual constraints in shaping lexical outcomes. More broadly, it underscores the need to conceptualise language development as an interaction between sensory input and cognitive subsystems, explaining why the comprehension–production gap is not a uniform feature of language acquisition.

## Summary

- Cross‐sectional data demonstrate a reduced lexical comprehension–production vocabulary gap specific to Williams syndrome, not observed in other populations.
- We present a computational model that reproduces the Williams syndrome lexical profile by combining domain‐general and visual‐specific processing constraints.
- The computational model explains how relatively strong lexical production can coexist with shallow semantic processing through exemplar‐based categorisation in Williams syndrome.
- This computational model links sensory processing, categorisation and lexical outcomes within a unified developmental framework.

## 1 Introduction

Language development is characterised by a striking and persistent asymmetry: comprehension consistently outpaces production, a phenomenon known as the *comprehension–production gap*. This gap appears across multiple linguistic domains—lexical, grammatical, phonological—and is observed universally across different languages and developmental time (e.g., Benedict 1979; Fenson et al. 1994; Gershkoff‐Stowe and Hahn 2013; Goldin‐Meadow et al. 1976; Hendriks and Koster 2010). Its pervasiveness raises a fundamental question: why do comprehension and production develop at different rates? In the lexical domain specifically, if a word has been acquired and integrated into an individual's vocabulary, why is it easier to comprehend than produce? While early in development, oro‐motor constraints partially explain the gap, they cannot fully account for its persistence into adulthood, as even typically developing adults continue to demonstrate this asymmetry (Gershkoff‐Stowe and Hahn 2013). This suggests the involvement of additional cognitive or computational processes. Here, we specifically focus on the differing computational demands of comprehension and production as potential drivers of this persistent asymmetry.

Computational models of word learning (Althaus and Mareschal 2013; Mayor and Plunkett 2010; McMurray et al. 2012; Plunkett et al. 1992) have elucidated key distinctions between two input modalities: visual and auditory. Comprehension involves a one‐to‐many mapping (word‐to‐object), while production demands a more challenging many‐to‐one mapping (object‐to‐word). For example, in comprehension, a child hearing the word *dog* might successfully associate it with a wide range of encountered dogs (e.g., different breeds, sizes and colours), demonstrating the one‐to‐many mapping. In contrast, during production, when shown a Golden Retriever, the child must retrieve the appropriate label *dog* from a broad set of possible words (e.g., animal, puppy, pet, doggy, Golden Retriever), illustrating the many‐to‐one challenge. Empirical patterns and methodological differences further emphasise this distinction: comprehension tasks often involve identifying the correct referent from a small set of images, whereas production tasks require generating the correct word from a much larger lexicon (McMurray et al. 2012). Moreover, this asymmetry reflects the statistical properties of input modalities: visual inputs vary considerably more than auditory inputs. For example, visual representations of an object such as a dog vary extensively in appearance (size, shape, colour), while the spoken word ‘dog’ remains comparatively stable in its phonological form. Consequently, production involves more competitive selection processes, increasing computational demands relative to comprehension. Notably, these considerations raise the possibility that differential constraints on visual and auditory processing may modulate the comprehension–production gap. Specifically, if a sensory modality develops atypically (i.e., yielding noisier or less reliable representations), this could affect lexical development and alter the expected gap.

The comprehension–production gap is nearly universal among typically developing individuals, though its magnitude may vary with linguistic environment and cross‐linguistic factors (Cattani et al. 2014; Siow et al. 2023). However, anecdotal reports suggest that this may not be the case for individuals with Williams syndrome, a genetic syndrome known for its uneven psychological profile, which includes heightened sociability and particularly weak visuospatial abilities (Farran et al. 2024; Pober 2010). Anecdotal reports suggest that in these individuals, typical comprehension–production asymmetry may be reduced, absent or even reversed. For instance, Bellugi et al. (2000) observed that while parents of children with Down syndrome (DS) often reported a pattern similar to typically developing children—where comprehension outpaces production—parents of children with WS described the opposite: ‘their children could say many words they did not understand’ (p. 11). These observations, including reports that language production is relatively strong compared to other developmental delays, have historically prompted claims of cognitive modularity in WS, proposing selective ‘sparing’ of language amidst broader cognitive impairments (Pinker 1999). However, more recent perspectives challenge this modular view, asserting instead that language is neither entirely spared nor can any neurodevelopmental conditions be accurately characterised by isolated domain‐specific impairments (D'Souza and Karmiloff‐Smith 2011).

Empirical studies of the comprehension–production vocabulary gap in WS remain inconclusive, partly due to methodological limitations such as small sample sizes (*N* < 14) or comparisons limited to individuals with DS (see Brock 2007, Mervis and Becerra 2007, for review). For example, Laing et al. (2002) found that 13 children with WS (aged 17–55 months) performed similarly to mental age matched typically developing children in both comprehension or production, whereas Van Den Heuvel et al. (2016) found that while 12 children with WS (aged 5–13 years) outperformed a group of 12 chronological age matched children with idiopathic intellectual disability on expressive vocabulary, they performed similarly on receptive vocabulary. In one rare large‐scale study on this topic, Mervis and Pitts (2015) found that 76 children with WS (aged 4–15 years) scored higher on standardised tests of language comprehension than on production (i.e., standardised to typically developing norms). However, when reassessed 3 years later, their standardised comprehension scores had declined more than their standardised production scores, though this interaction with time was not statistically significant. These mixed findings underscore the need for robust empirical investigation to clarify the nature of the comprehension–production relationship in WS.

In the present study, we empirically examine the lexical comprehension–production vocabulary gap in a substantial cohort of young children with WS, spanning early development from 7 months to 6 years of age. We situate this profile within a broader developmental context by including cross‐syndrome comparisons with children with DS and fragile X syndrome (FXS), as well as contrasts with typically developing (TD) children. Although vocabulary delays are common across neurodevelopmental conditions, the nature of these delays—and potentially the relationship between comprehension and production—varies markedly by syndrome. In DS, comprehension typically exceeds production, with expressive language often lagging behind receptive vocabulary (Mason‐Apps et al. 2020). This pattern mirrors the canonical comprehension–production asymmetry observed in typical development. FXS, by contrast, is associated with wide variability in language outcomes, often featuring pronounced expressive delays exacerbated by attentional and behavioural difficulties –though studies involving young children with FXS remain relatively rare (Brady et al. 2006). WS presents a particularly distinctive linguistic profile: despite global developmental delays, anecdotal and clinical reports have long noted that expressive vocabulary appears unusually strong relative to overall cognitive functioning (Bellugi et al. 2000). These divergent profiles offer a window into how lexical organisation develops under varying constraints.

The atypically developing groups included children up to 6 years of age to capture the broader developmental window during which children with genetic syndromes typically acquire early receptive and expressive vocabularies. In contrast, the TD group was capped at 25 months to reflect the typical age at which children acquire vocabularies comparable in size to those of older children with WS. This also aligns with the standard age range for the Oxford Communicative Development Inventories (CDI), a standardised parent‐report questionnaire used to assess comprehension and production vocabulary sizes in children up to 25 months. Our primary aim was to match the groups on vocabulary size rather than chronological age, in order to examine whether the comprehension–production gap differs between groups at comparable vocabulary levels, despite differences in age. Matching on age would have confounded this comparison, as children with WS typically reach language milestones much later than their TD peers.

We complement this empirical analysis with computational modelling using self‐organising maps (SOMs), a powerful tool for simulating developmental cognitive processes, especially relevant given known sensory atypicalities in WS, including pronounced visuospatial difficulties (Farran et al. 2024; Thom et al. 2023). Furthermore, the computational modelling approach allows us to test the hypothesis that the atypical developmental trajectory of language acquisition in children with WS is related to categorisation abilities that have been described as atypical (Nazzi and Bertoncini 2003), weaker than those of typically developing children (Purser et al. 2011), or even absent (Nazzi and Karmiloff‐Smith 2002). Children with WS are thought to rely on relatively strong speech perception and memory skills to acquire a large proto‐lexicon, gradually forming associations between specific sound patterns and individual exemplars of a category, rather than abstracting over them (Nazzi and Bertoncini 2003). Consequently, atypical categorisation in WS may lead to difficulties in word‐object generalisation (Stevens and Karmiloff‐Smith 1997) and reduced semantic access (Bellugi et al. 2000; Thomas and Karmiloff‐Smith 2003), possibly accounting for the reduced comprehension–production gap (Paterson 2000; Harris et al. 1997).

To explore these possibilities, we tested multiple computational implementations with different processing constraints to determine which best captured the empirical data. To this end, we manipulated three key properties of the SOM: (a) map size; (b) input noise; and (c) the neighbourhood function, to explore their relevance in shaping lexical processing in WS. These components were chosen based on their relevance to both SOM function and the cognitive processing difficulties characteristic of WS. Reducing map size constrains computational capacity, limiting efficient information encoding; introducing input noise simulates representational uncertainty and sensory unreliability; disrupting the neighbourhood function impedes cluster formation crucial for categorisation processes. By evaluating these distinct computational constraints, we aim to elucidate the mechanisms underlying lexical comprehension–production dynamics observed in WS, thereby providing broader insights into language development processes.

## 2 Methods

### 2.1 Empirical Study

#### 2.1.1 Participants

Vocabulary data from a total of 1319 children were analysed using parental reports collected via the Oxford Communicative Developmental Inventories (Oxford CDIs; Hamilton et al. 2000). The sample included 67 children with Williams syndrome (WS; 28 girls, mean age = 29 m 22 d, median = 28 m 5 d, range = 7 m 15 d–73 m 17 d), 27 children with Down syndrome (DS; 15 girls, mean age = 22 m 18 d, median = 22 m 17 d, range = 11 m 27 d–39 m 8 d) and 15 children with fragile X syndrome (FXS; 3 girls, mean age = 31 m 18 d, median = 32 m 6 d, range = 14 m 23 d–46 m 6 d). All participants resided in the United Kingdom at the time of data collection. The parents spoke only or mostly English to their children. One exception was the mother of a child with WS, who noted that she was teaching some French to her child. While some children may have had exposure to other languages (e.g., through extended family or community settings), such exposure is expected to have been minimal.

Additionally, data from 1210 monolingual, typically‐developing (TD) children (559 girls, mean age = 18 m 23 d, median = 18 m 0 d, range = 12 m 0 d–25 m 0 d) were obtained from the UK‐based dataset (Floccia 2017), a subsection of the WordBank database (Frank et al. 2017). This dataset includes only monolingual children and was selected to ensure consistency in linguistic and cultural context across groups.

Data from children in the WS, DS and FXS groups were collected with informed parental consent, and procedures were approved by the Ethics Committee of the Department of Psychological Sciences at Birkbeck, University of London, and by the UK National Health Research Authority, in accordance with the Declaration of Helsinki.

#### 2.1.2 Materials

Vocabulary was assessed using the Oxford CDI (Hamilton et al. 2000), a parental‐report measure containing 418 words across 19 semantic categories (e.g., body parts, household items, action words). Receptive vocabulary scores represented the number of words parents reported their child understood, whereas expressive vocabulary scores included only words the child both understood and produced.

#### 2.1.3 Analytical Approach

**FIGURE 1:**
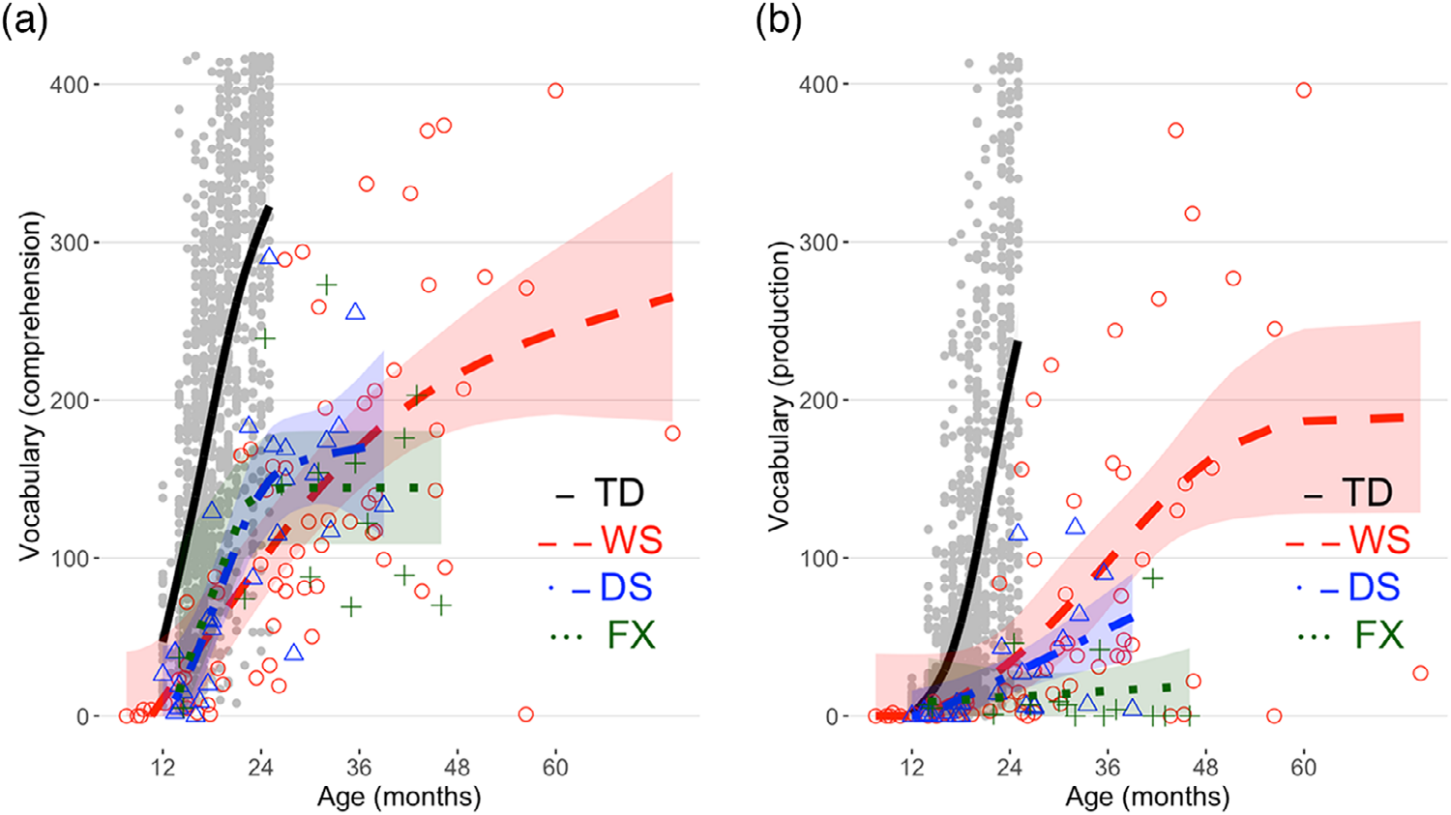
Comprehension (a) and production (b) delays in Williams syndrome (WS), Down syndrome (DS) and fragile X syndrome (FXS) compared to typically developing (TD) children. Shaded areas correspond to the 95% CIs (undistinguishable for TD).

**FIGURE 2:**
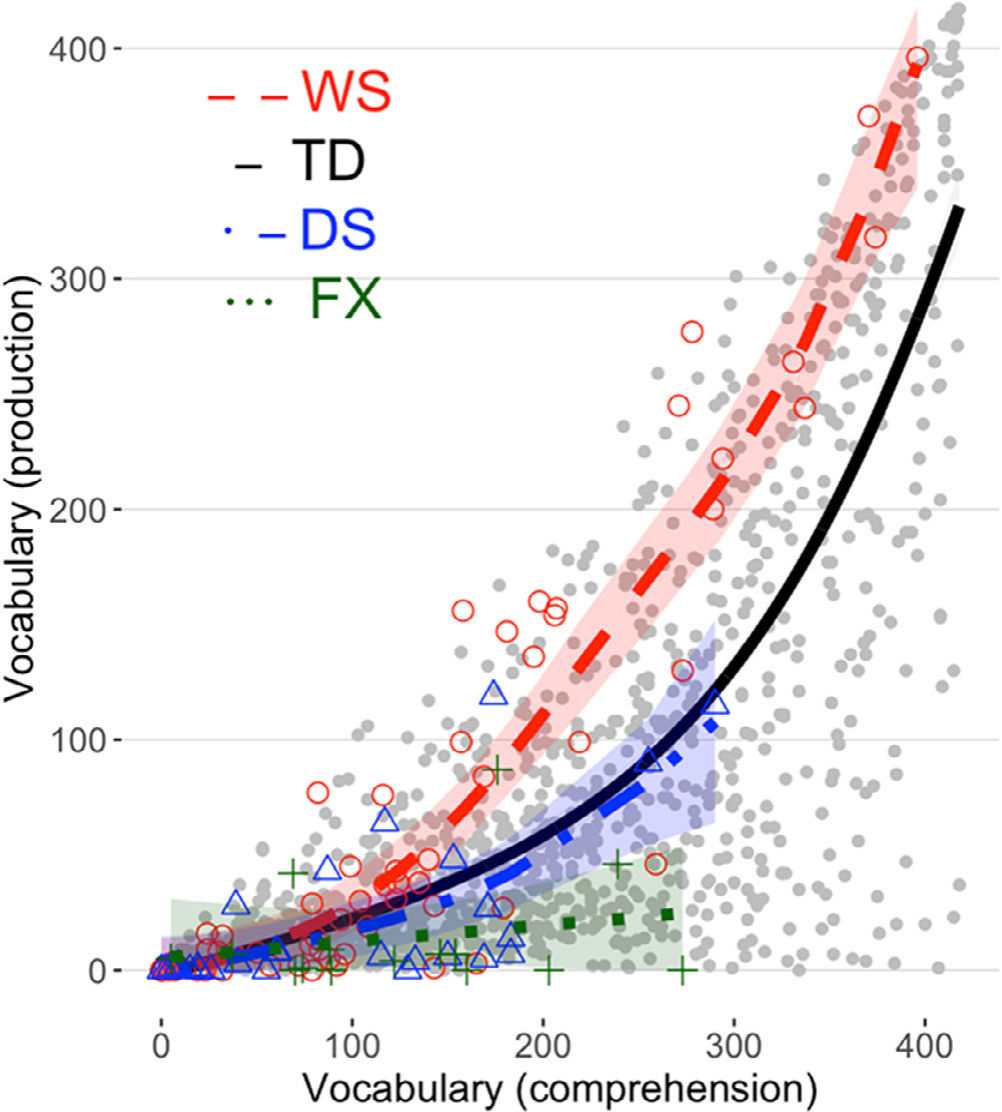
Williams syndrome (WS) shows a reduced comprehension–production asymmetry compared to typically developing (TD) children and Down syndrome (DS), while fragile X syndrome (FXS) demonstrates overall reduced production. Shaded areas correspond to the 95% CIs (undistinguishable for TD).

We applied shape‐constrained additive models (SCAM, Pya and Wood 2015) using the *scam* R package (Pya 2025) to estimate vocabulary comprehension (Figure 1a) and production (Figure 1b) across age, implementing a monotonic positive increase (*mpi*) constraint to reflect expected developmental growth. Models were fitted with a basis of *m_basis = 3* to account for expected smoothed growth patterns. Model predictions and 95% confidence intervals were generated via the *predict* function. We adopted the same approach to model vocabulary production as a function of vocabulary comprehension (Figure 2).

#### 2.1.4 Empirical Results

Visual inspection of Figure 1a,b is sufficient to assert that the vocabulary size of each individual in the syndrome groups, in both comprehension and production, lags behind the typical TD vocabulary growth (solid black line).

To determine whether WS participants display a reduced comprehension–production gap, we conducted an exact binomial sign test to assess whether WS data points were significantly above the TD prediction line (Figure 2). This test evaluated whether the median of the residuals (the differences between observed WS production values and predicted TD production values) was significantly greater than zero. The analysis revealed that of the 67 WS data points, 47 had positive residuals (i.e., above the TD prediction line), with a probability of positive residuals of 0.701 (CI = [0.596, 1.000], *p* < 0.001), indicating WS production scores were statistically above the TD predictions. Exact binomial tests for DS (9 above, 18 below, probability of positive residual = 0.33, CI = [0.186, 1.000], *p* = 0.974) and FXS (4 above, 11 below, probability of positive residual = 0.27 (CI = [0.097, 1.000], *p* = 0.982) were not significant. To summarise, the reduced comprehension–production vocabulary gap appears specific to WS.

### 2.2 Computational Model

#### 2.2.1 Model Overview

**FIGURE 3:**
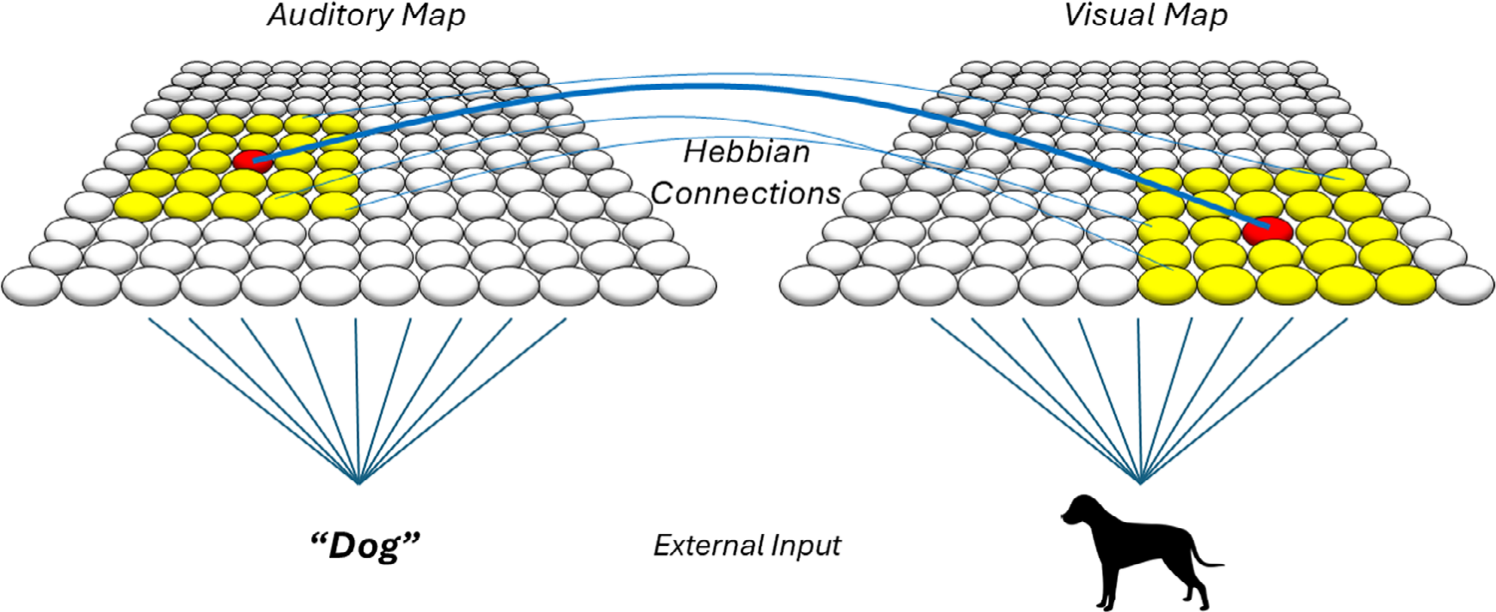
Architecture of the computational model. Two self‐organising maps (auditory and visual) were linked via Hebbian learning, facilitating structured category representations and cross‐modal mappings. Only a subset of Hebbian connections is visualised for clarity, due to the full connectivity making the figure visually dense.

The computational model was based on the assumption that lexical acquisition involves forming auditory and visual categories and establishing bidirectional mappings between them (Althaus and Mareschal 2013; Mayor and Plunkett 2010; McMurray et al. 2012; Tovar et al. 2020). It focused on simple, concrete object‐word pairings, without addressing abstract or relational vocabulary, which may develop under different constraints. The model consisted of two SOMs, one processing auditory inputs and the other visual inputs. These maps were fully connected through associative Hebbian learning (Figure 3), which enables cross‐modal mappings following Hebb's rule: *neurons that fire together wire together*.

SOMs are neural networks in which neurons are arranged in a two‐dimensional grid and learn to categorise patterns without external supervision (Kohonen 2013). To do this, each individual neuron can represent input stimuli—neurons have the same dimensionality such that their weight vector can match that of the input stimuli. In this way, weight vectors represent specific combinations of features that serve as internal representations of external stimuli. A SOM can thus be understood as a collection of structured representations of the external world. In short, each neuron starts with a vector of random value. Exemplars (e.g., a word form or an object token) are fed into the SOM one at a time as input vectors. Each time an exemplar is introduced to the SOM, the neuron whose current value most closely matches the data (the ‘winning’ neuron—or Best Matching Unit [BMU]), and its neighbours on the map, are activated and update their values incrementally, becoming more similar to the data point. Early in training, activation spreads broadly across the map, with larger regions responding to each exemplar. As learning progresses, the activation radius narrows and neurons become more specialised. Over time, this repeated adjustment leads to topographically‐organised maps, where similar exemplars cluster together in nearby areas, and dissimilar ones are mapped farther apart.

We designed a Reference Model to serve as a baseline implementation capturing typical development, determining an efficient number of neurons for category formation and an input variability level that consistently yields a comprehension advantage over production. In this model, each SOM consisted of 144 neurons arranged in a 12 × 12 grid (Figure 3). Initially, neurons were assigned random weight vectors, meaning that the system had no predefined categories or prior knowledge of the input. Both maps were structurally identical. Their differentiation into auditory and visual maps emerged solely from the statistical properties of the input each received.

#### 2.2.2 Training and Testing

Learning in the model involved two concurrent processes:

1. Category formation: Within‐modality self‐organisation, where similar stimuli activate neighbouring neurons, creating topographically structured categories on the SOMs.
2. Auditory‐visual association: Hebbian learning strengthened connections between co‐activated auditory and visual neurons, leading to associations that emerge at the category level.

Training involved 400 epochs with 20 words, each presented as eight exemplars per category in each modality (auditory and visual; 160 unique input patterns per modality; see Section 1.4 of the Supporting Information for further details). Each epoch included exposure to all category exemplars. To simulate realistic exposure to auditory‐visual pairings, training incorporated variability throughout familiarisation (e.g., hearing ‘*dog*’ pronounced by different speakers while viewing different dog breeds).

After each training epoch, we tested lexical comprehension and production. In comprehension trials, an auditory label was presented to the model in isolation, and we examined whether the resulting activation pattern in the visual map corresponded to the correct object. In production trials, one of the visual exemplars was presented alone, and we assessed whether the model activated the corresponding auditory label associated with that object. We tracked these performance measures across epochs to examine the emergence of the comprehension–production gap.

In addition to evaluating lexical development, we assessed how well the model processed exemplars and formed internal representations. Given that the comprehension–production gap may be shaped by the reliability of sensory representations, we included two metrics to quantify these properties: Quantisation Error (QE) and categorisation metrics. QE reflects how well the model captures the structure of the input. Lower QE values indicate internal representations that match the input more closely. To evaluate categorisation, we identified the BMUs activated for each exemplar and analysed the total number of distinct BMUs by the end of the final training epoch. Given that the vocabulary consisted of 20 words, a categorisation with exactly 20 BMUs indicates that all exemplars of a category (e.g., different dog breeds) are mapped to a single representation, suggesting a prototype‐based organisation. In contrast, a larger number of BMUs reflects more dispersed category representations, where multiple locations on the map process exemplars from the same category, which aligns with exemplar‐based representations, where objects are mapped individually rather than under a common prototype. To ensure comparability across models, BMU counts were normalised relative to the total number of neurons available for selection, providing a standardised measure of category compactness in the range [0, 1]. Higher values indicate exemplar‐based processing, while lower values suggest prototype‐based categorisation, as fewer BMUs reflect greater abstraction across exemplars. All model equations, learning rules and testing procedures are detailed in Section 1 of the Supporting Information.

#### 2.2.3 Stimulus Set Design and the Comprehension–Production Gap

To investigate how modality‐specific input variability might shape the comprehension–production gap, we constructed a simplified artificial vocabulary comprising 20 symbolic ‘word’ labels paired with corresponding ‘object’ categories. These were not real words or referents but were represented as arbitrarily generated 10‐dimensional vectors. This abstraction enabled us to systematically manipulate the statistical structure of the input while holding other factors constant (see Section 1.4 of the Supporting Information for details).

By using symbolic input rather than existing words, we were able to isolate the effects of within‐category variability in auditory and visual modalities—specifically, how differences in this variability between input modalities influence the emergence of the comprehension–production gap. This design choice also avoided potential confounds associated with lexical frequency, familiarity or semantic richness.

To capture natural variability within categories, exemplars were generated around their category prototypes—whereby variations around visual prototypes (*σ* = 0.25) were larger than in the auditory domain (*σ* = 0.05). These values were initially set when establishing the Reference Model, as they consistently led to a comprehension advantage over production, a pattern aligned with the typical gap in lexical development. Moreover, this distinction was made on the assumption that visual objects (e.g., different dog breeds) exhibit greater within‐category variation than their corresponding spoken labels. Although this may not always be the case, visual inputs are arguably often more variable than auditory word forms, where visual referents like ‘dog’ may be encountered in diverse visual formats (across size, shape, colour, posture, movement, texture; across lighting, angle, context; as photographs, cartoons, plush toys, etc.), while the word itself must maintain a relatively and appropriately stable phonological form (/d/ /ɔ/ /g/) to be learnable. Prior neurocomputational models have demonstrated that this statistical asymmetry plays a central role in shaping the comprehension–production vocabulary gap (Althaus and Mareschal 2013; McMurray et al. 2012; Mayor and Plunkett 2010). Furthermore, this asymmetry is also observed in models addressing referential ambiguity in word learning (McMurray et al. 2012). These models simulate situations in which the learner hears a single word (e.g., ‘dog’) while observing a scene with multiple objects (e.g., a dog, a ball and a tree), and must associate the word with the correct object. Across trials, the same word is paired with different sets of visual competitors. Computationally, this one‐to‐many mapping (from auditory to visual) across trials with varying visual competitors can be conceptualised as low variability in auditory input and high variability in visual input, reinforcing the role of differential input variability in shaping lexical acquisition dynamics.

#### 2.2.4 Modelling Williams Syndrome

To investigate which processing constraints might reproduce the empirical WS profile, we implemented and compared multiple variations of the model. Specifically, we aimed to identify configurations that simultaneously captured (1) an overall lexical developmental delay and (2) a reduced comprehension–production gap relative to the Reference Model. To this end, we examined the effects of manipulations to three key components of the model:

1. SOM size reduction: Decreasing neuron count limits representational capacity and constrains the learning space. Map size was reduced from 12 × 12 (144 neurons) in the Reference Model to 9 × 9 (81 neurons) in the tested models, based on pilot simulations showing that smaller maps consistently led to delayed learning trajectories.
2. Input noise: Adding noise to input vectors affects representation consistency, simulating sensory or representational difficulties. This manipulation is intended to simulate reduced perceptual fidelity or noisier encoding of sensory information. This aligns with evidence of atypical sensory processing in WS, which could disrupt the stability of category learning. Details of the noise implementation, including its magnitude and structure, are provided in Section 1.6 of the Supporting Information.
3. Neighbourhood disruptions: Disrupting the neighbourhood function limits how much neighbouring neurons adjust their weights in response to a stimulus, reducing the network's ability to generalise across similar inputs. This results in narrower, less integrated category structures and reflects a breakdown in the spatial organisation of representations on the map.

For full implementation details and parameter values, see Section 1 of the Supporting Information.

To systematically assess how the perturbations in these components shape lexical development, we first examined their individual effects within each sensory modality, on developmental delays and comprehension–production gap reduction. We then explored integrated models combining multiple perturbations across modalities, leading to a total of 17 distinct models to identify the configuration that best approximated the empirical WS trajectory. Specifically, we tested each of the three manipulations (map size reduction, input noise and neighbourhood disruption) independently in the auditory and visual modalities (3 × 2 = 6), as well as a cross‐modal version of each (3 additional models), yielding 9 models. The remaining 8 models involved combinations of two or more manipulations, informed by the performance patterns observed in the initial set of 9 models. The full set of model configurations is provided in Section 2 of the Supporting Information.

### 2.3 Modelling Results

Each model configuration was run 20 times independently to ensure robust performance assessment. The complete analysis of all models is available in Section 2 of the Supporting Information. Here, we focus on the results of the selected WS model.

To determine whether a model approximated the lexical profile of WS, we focused on the two key criteria which had to be simultaneously met: (1) delayed lexical acquisition and (2) a reduced comprehension–production gap. Lexical delays were quantified using the mean area under the curve (AUC) for comprehension and production trajectories across training epochs. The AUC provides a cumulative measure of learned words over time, reflecting the total number of words accurately processed throughout training. We computed deltas by subtracting the AUC of the Reference Model from that of each tested model; negative values indicate delays. To assess the comprehension–production gap, we mirrored the analysis used for the empirical data: a binomial sign test was applied to determine whether each model's production values fell significantly above the Reference Model's prediction line. For this, we randomly selected 5 data points from each of the 20 simulation runs per model configuration, yielding a total of 100 data points per model. Full statistical results and significance values are provided in Section 2 of the Supporting Information.

**FIGURE 4:**
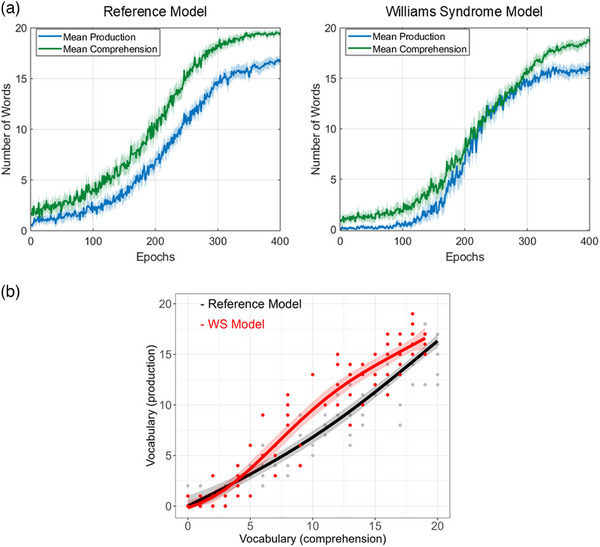
Panel (a) shows the developmental trajectories of comprehension and production for both the Reference Model and the Williams syndrome Model. Panel (b) shows the relationship between comprehension and production. To align with the empirical analysis, we applied the same approach used for the empirical data, using shape‐constrained additive models (SCAM) with a monotonic positive increase (*mpi*) constraint and *m_basis = 3* for smoothing. For each of the 20 independent runs of each condition (Reference Model and WS), we randomly selected 5 data points to be plotted, each representing comprehension and production levels at different stages of training, and the regression (SCAM) curve was superimposed to capture developmental trends, highlighting the WS model's relative production advantage. Shaded areas correspond to the 95% Cis.

Among all tested configurations, just one specific model (i.e., henceforth referred to as the WS model) successfully replicated the characteristic WS lexical profile by meeting both criteria: that comprehension and production were delayed in this model, as evidenced by lower AUC values compared to the Reference Model (*Δ*comp = −770.05, *t *= −32.82, *p*(FDR) < 0.001; *Δ*prod = −191.30, *t *= −6.7, *p*(FDR) < 0.001), and with a reduced comprehension–production gap, with 62 out of 100 data points falling above the level predicted by the Reference Model (*p* = 0.01, 95% CI = [0.53, 1]). The learning trajectories for comprehension and production for both the Reference Model and the WS Model are presented in Figure 4a, while Figure 4b displays the regression (SCAM) fits of production levels as a function of comprehension. Summary results for the remaining models that did not meet both criteria are presented in Section 2 of the Supporting Information. Notably, the WS Model included a combination of domain‐general and modality‐specific perturbations: (a) reduced map sizes in both auditory and visual networks; (b) increased noise in both input modalities; and (c) impaired neighbourhood function specifically in the visual map.

Our analysis, based on comparing all simulations (see Section 2 of the Supporting Information), revealed that the three model manipulations affected lexical performance in specific ways. Reducing the size of either the auditory or visual map resulted in lexical delays, affecting both comprehension and production. However, decreasing the size of the visual map had a more pronounced impact on production, while reducing the auditory map primarily delayed comprehension, reflecting interactions with modality‐specific differences in input variability (see below). Increased noise in either input modality also led to delays with a similar pattern, visual noise had a stronger effect on production, while auditory noise had it on comprehension. While these two perturbations had a similar delaying effect on lexical acquisition, neither contributed to reducing the comprehension–production gap.

Disruptions to the neighbourhood function had more nuanced effects on lexical performance, with auditory and visual perturbations differing considerably. A visual neighbourhood disruption alone caused a dramatic reduction of the comprehension–production gap, driven by increases in production, also to a lesser extent, comprehension. In contrast, an auditory neighbourhood disruption had a much smaller effect, only slightly increasing comprehension without driving production upwards. These modality‐specific effects likely result from interactions with input variability, as the only distinction between the visual and auditory maps was the degree of variability in their inputs. This highlights the role of interactions between processing constraints and input regularities in shaping the lexical outcomes.

Among the models exhibiting lexical delays, the selected WS Model showed the smallest comprehension–production vocabulary gap (WS model gap; AUC comprehension—AUC production = 548.98; Reference Model gap = 1127.73) and was the only model to show a significant result in the binomial sign test. Figure 4 shows how the WS Model and the Reference Model follow distinct lexical acquisition trajectories. Figure 4b, particularly highlights a shift in the interaction between comprehension and production that characterises the WS lexical profile.

### 2.4 Connecting Basic Cognitive Processes With Language Outcomes: Insights From QE and Categorisation Metrics in the Model

**FIGURE 5:**
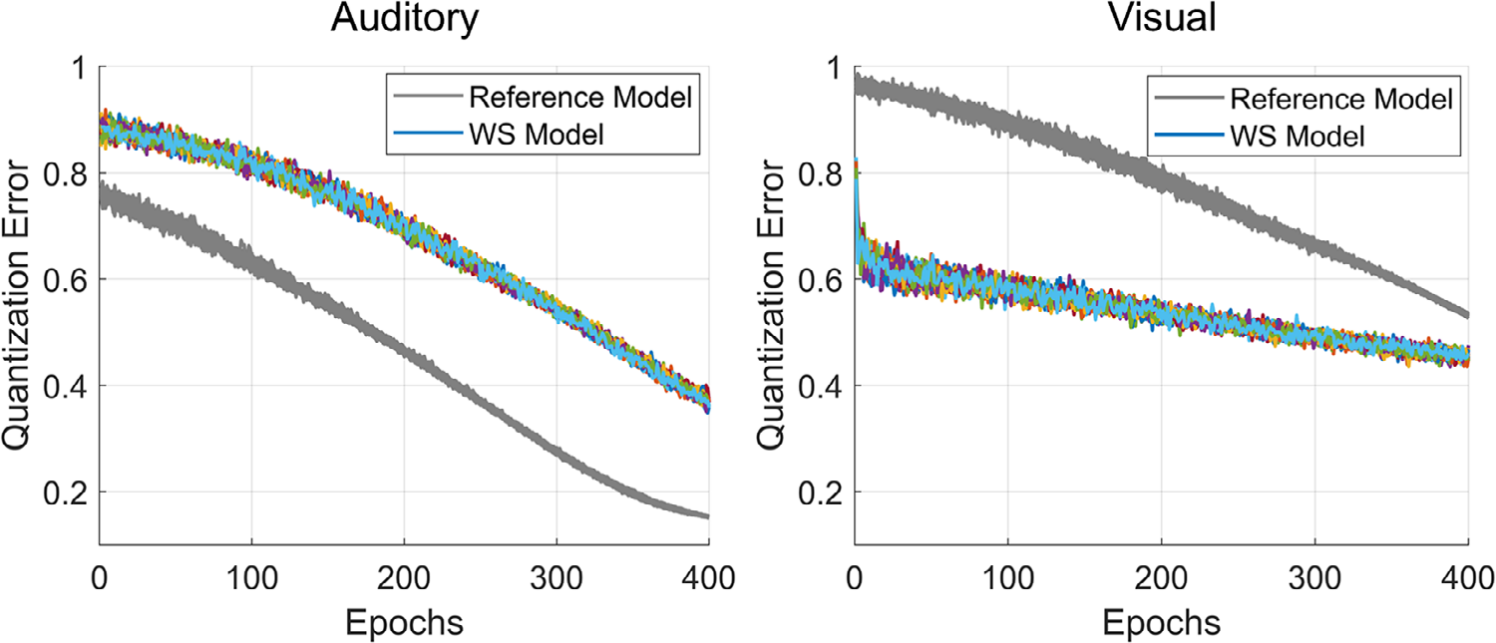
The left panel shows the Quantisation Error (QE) evolution in the auditory domain for both the Reference Model (grey lines) and the WS Model (coloured lines), with all 20 runs of each model overlaid. The right panel presents the same analysis for the visual domain. Higher QE values reflect less precise internal representations. The WS Model exhibits a delayed trajectory in the auditory domain. However, in the visual domain, it shows an atypical trajectory characterised by overall lower QE values but increased fluctuations, indicating that visual representations remain inflexible over time.

Having identified the WS Model, we further analysed the development of its auditory and visual maps, revealing surprising patterns through QE and categorisation metrics, which pointed to both delayed and atypical learning dynamics. As seen in Figure 5, in the WS simulations, auditory QE was consistently delayed relative to the Reference Model, suggesting a slower refinement of representations in the auditory domain. However, when considering the visual QE, it exhibited early low values but with high variability, indicative of visual representations that were specific but inflexible.

**FIGURE 6:**
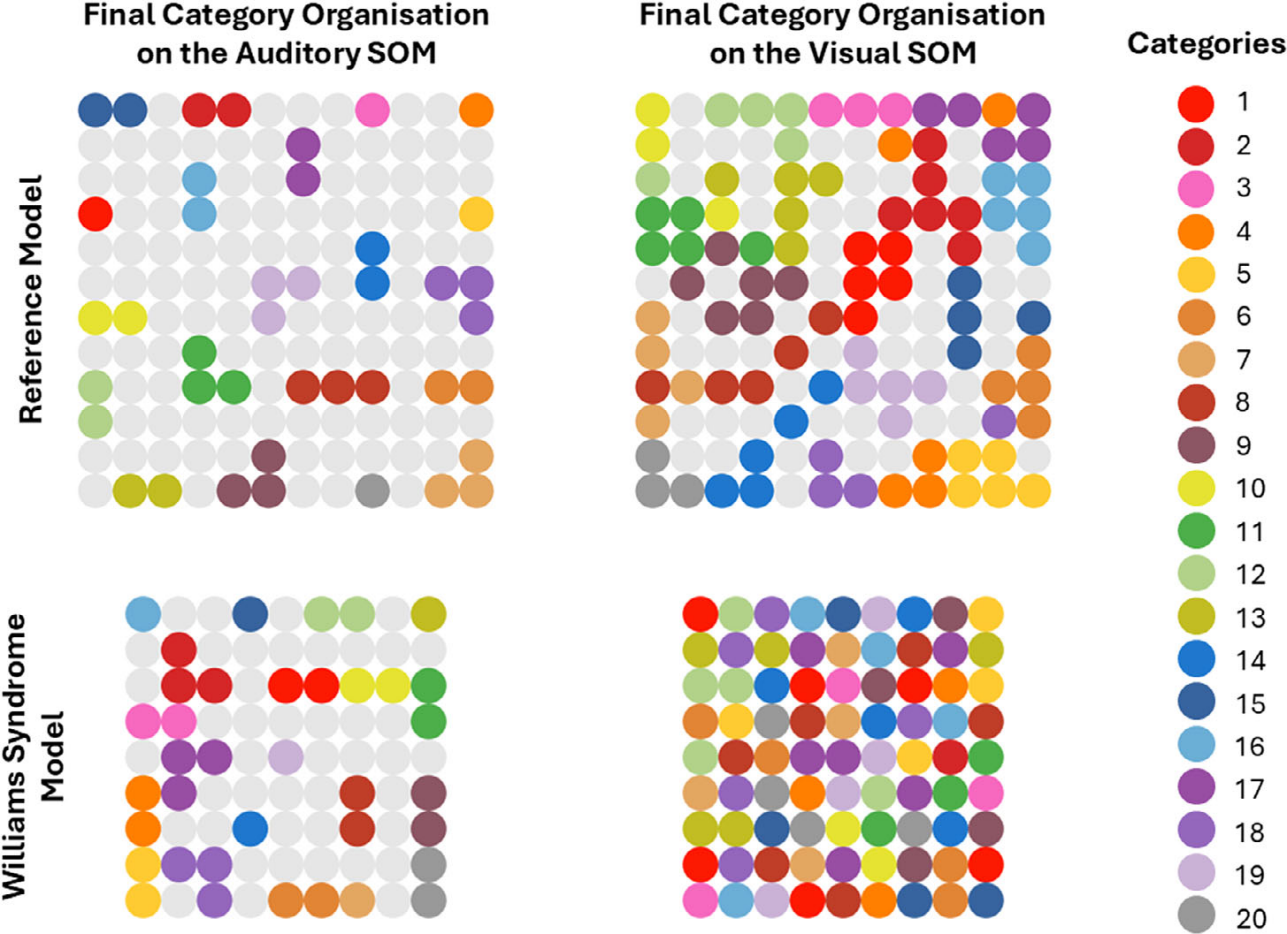
Category organisation on the auditory and visual SOMs for the Reference and WS models. Each colour represents a category, with BMUs shown in the corresponding colour. In the Reference Model (upper row), categories are organised into well‐defined clusters, with clear separation and within‐category compactness, particularly in the auditory SOM. In the Williams syndrome Model (lower row), auditory categorisation remains structured, but the visual SOM shows severe disruption; BMUs for each category are scattered across the map, lacking compactness and clear organisation, aligning with an exemplar‐based representation. As described in the main text, the WS SOMs are smaller than those in the Reference Model to account for lexical delays.

This instability in visual representations was further reflected in the categorisation metric, where the WS Model displayed the highest number of distinct BMUs (WS Model visual category rating *M* = 1, *SD* = 0.0; auditory category rating: *M* = 0.412, *SD* = 0.03; Reference Model visual category rating *M* = 0.676, *SD* = 0.02, auditory category rating *M* = 0.282, *SD* = 0.01). Since this metric ranges from 0 to 1, with higher values indicating more exemplar‐based representations and lower values reflecting more compact, prototype‐based categorisation, these results suggest that the WS model exhibited a markedly fragmented visual categorisation. While the auditory categorisation rating remained comparable to the Reference Model, the visual SOM in the WS Model failed to consolidate exemplars into shared category representations, instead processing each instance in a more isolated manner. This effect is also evident in Figure 6, which displays the final organisation of categories on the SOMs. Each SOM shows all its neurons, with coloured ones indicating BMUs that processed exemplars from different categories. Each colour represents a distinct category, and its position reflects where that category was mapped. In the Reference Model, categories are grouped into compact clusters, meaning exemplars of the same category were consistently processed in nearby locations of the neural network. In contrast, the WS model exhibits a dispersed, collage‐like distribution of colours, where exemplars from the same category are processed in distant locations rather than forming cohesive clusters. This disorganised mapping suggests difficulties in category formation, aligning instead with exemplar‐based representations.

Taken together, these results suggest that the WS Model was learning from individual instances rather than forming generalisable category structures. Unlike the Reference Model, in which different exemplars of the same category were mapped under a shared representation, the WS Model struggled to connect one instance to another, instead treating exemplars as isolated objects.

## 3 Discussion

Our study presents the first systematic investigation of the comprehension–production gap in WS, combining empirical data with computational modelling to elucidate the possible underlying mechanisms that shape this distinct linguistic profile. Our empirical data demonstrated that children with WS indeed show a reduced comprehension–production gap compared to typically developing (TD) children and two other neurodevelopmental conditions (DS and FXS), establishing the specificity of the WS profile and confirming previously anecdotal observations. More importantly, our computational model provides a possible mechanistic account of how this lexical pattern can emerge from a combination of domain‐general and modality‐specific constraints, with atypical visual processing playing a central role in reducing the comprehension–production gap.

### 3.1 The Comprehension–Production Gap in WS

Our empirical analysis revealed that, similarly to the other conditions, children with WS show overall delays in vocabulary development relative to TD children. However, their comprehension–production asymmetry is significantly reduced. Unlike TD and other neurodevelopmental groups, participants with WS exhibited a disproportionately higher production vocabulary given their level of comprehension, aligning with previous anecdotal and small‐scale studies (e.g., Bellugi et al. 2000; Van Den Heuvel et al. 2016). However, our study is the first to systematically quantify this effect across a large cross‐syndrome sample.

This distinctive lexical profile calls into question the assumption that the comprehension–production asymmetry is a universal feature of language development. Our modelling results demonstrate how the relationship between comprehension and production is not fixed but emerges from interactions between learning restrictions and sensory input. Moreover, our results raise important theoretical questions about why WS deviates from the canonical trajectory of language development.

### 3.2 The Role of Visual Processing in Vocabulary Development

Our computational modelling results identify a specific combination of processing perturbations that successfully replicates the WS lexical profile: (a) reduced map sizes in both auditory and visual networks; (b) increased noise in both input modalities, and—most critically—(c) impaired neighbourhood function specifically in the visual map. While the first two perturbations contributed to general lexical delays, only the visual‐specific neighbourhood manipulation reduced the comprehension–production gap. This finding suggests that the distinctive lexical profile in WS, characterised by a reduced comprehension–production gap, emerges primarily from atypical visual processing rather than from enhanced verbal abilities or auditory processing advantages.

This exploration led us to consider that accurately modelling WS required combining delays caused by generalised auditory and visual perturbations with the relative production advantage driven by visual‐specific constraints. This configuration proved particularly compelling given the extensive literature on visuospatial difficulties in WS (e.g., Farran et al. 2024; Thom et al. 2023), as it allowed us to integrate well‐established cognitive characteristics into a mechanistic account of lexical development.

Our model goes beyond simply acknowledging visuospatial constraints in WS, to explain how these may mechanistically shape language outcomes. The model that best captures WS‐like behaviour exhibits learning properties consistent with exemplar‐based processing, potentially driven by visual neighbourhood perturbations, where individual instances are encoded without forming broader categories. This results in item‐specific word‐object mappings, rather than prototype‐based generalisations. Categorisation metrics and BMU visualisations support this interpretation: the WS model showed the most dispersed BMU patterns, particularly in the visual domain, indicating difficulty in forming coherent category structures.

This mechanism may help explain why individuals with WS often produce words fluently while struggling with conceptual understanding (Bellugi et al. 2000). Their learning may rely more on direct associative mappings than on integrated semantic networks, consistent with longstanding and current descriptions of shallow semantic representations despite a relative strength in vocabulary (Romero‐Rivas et al. 2023; Romero‐Rivas et al. 2025; Thomas and Karmiloff‐Smith 2003).

Our model thus provides a computational tool that may help to connect visual processing, categorisation mechanisms and lexical outcomes within a unified developmental framework. Moreover, these simulations highlight the complex interplay between different cognitive processes in lexical development. Rather than viewing the reduced comprehension–production gap in WS as evidence for modular language skills that develop independently of general cognition (Pinker 1999; Piattelli‐Palmarini 2001), our findings suggest that this linguistic profile emerges directly from domain‐general processing constraints, particularly in the visual modality.

### 3.3 Rethinking the Comprehension–Production Gap

The current findings have broader implications for theories of language development and the comprehension–production relationship. While a comprehension–production gap remains present in WS, its reduced magnitude challenges the notion that the typical form of this asymmetry is a universal and invariant feature of language development. Instead, our results support a more dynamic perspective, in which the gap emerges from domain‐general processing constraints that may vary across developmental contexts. Moreover, they underscore the importance of the interaction between internal processing constraints and environmental regularities, just as studies in bilingual populations have shown that variations in input structure can modulate the magnitude of the gap (Cattani et al. 2014; Siow et al. 2023).

Our model also demonstrates how constraints in non‐linguistic domains, particularly visual processing, can reshape the relationship between comprehension and production through their effects on internal representational structures. While most accounts emphasise auditory and articulatory factors, our study suggests that the structure of visual input plays a critical role. Future research should investigate whether similar effects occur in other populations with atypical sensory processing, such as autism.

### 3.4 Implications

Our findings have direct implications for interventions targeting language development in WS. If the reduced comprehension–production gap stems from exemplar‐based visual processing, interventions might focus on supporting the development of more coherent categorical representations. This could involve structured categorisation activities that explicitly highlight similarities between exemplars of the same category, potentially strengthening conceptual understanding while building on existing production strengths.

The observation that production may outpace conceptual understanding in WS raises the possibility that vocabulary assessments relying solely on expressive measures could overlook gaps in semantic knowledge. Some children with WS might use words without fully understanding their meanings, pointing to the potential value of more comprehensive assessment strategies that consider both expressive use and underlying conceptual understanding. Practitioners and caregivers may need to consider that expressive vocabulary in WS does not always reflect depth of understanding, and that more nuanced evaluation approaches could provide a clearer picture of linguistic competence.

More broadly, our findings highlight the importance of considering domain‐general processing constraints when developing language interventions for neurodevelopmental conditions. Rather than targeting linguistic skills in isolation, effective interventions might address the underlying processing mechanisms that shape language outcomes, such as visual categorisation and cross‐modal integration.

### 3.5 Limitations and Future Directions

While our study provides novel insights into the lexical profile of WS, some limitations should be acknowledged. First, our empirical data relied on parental reports, which, while widely validated, may be subject to reporting biases. Future studies should complement these measures with direct assessments of comprehension and production. Second, our computational model, while biologically inspired and capturing key aspects of lexical development, represents a simplified approximation of real‐world language learning. Future work should explore more complex neural architectures, incorporating motor, social, attention and memory constraints.

## 4 Conclusion

Our study provides the first systematic empirical evidence for a reduced comprehension–production gap in WS and identifies specific processing constraints that may contribute to this distinctive lexical profile. By combining empirical data with computational modelling, we demonstrate that the atypical relationship between comprehension and production in WS can emerge from domain‐general processing constraints, particularly in visual categorisation. Furthermore, we propose a novel mechanistic account: selective visual processing difficulties lead to an exemplar‐based learning strategy, driving the atypical lexical profile observed in WS. These findings not only enhance our understanding of language development in WS but also offer broader insights into how sensory constraints interact to shape lexical outcomes.

Crucially, this approach allows us to map potential mechanistic pathways underlying lexical development in WS, leveraging the model to infer which computational constraints are most critical for shaping atypical learning trajectories. By systematically testing different perturbations, we move beyond descriptive characterisations of WS language profiles, providing a framework that links domain‐general cognitive constraints to lexical outcomes. This methodology aligns with a broader computational perspective on neurodevelopmental conditions, demonstrating how modifications to domain‐general mechanisms can lead to specific developmental phenotypes.

## Author Contributions

**Dean D'Souza**: writing – review and editing, writing – original draft, conceptualization, methodology, investigation, data curation, project administration, supervision. **Hana D'Souza**: conceptualization, writing – review and editing, investigation, writing – original draft, supervision. **Julien Mayor**: conceptualization, investigation, methodology, software, data curation, supervision, writing – review and editing, visualization, formal analysis, project administration. **Ángel Eugenio Tovar**: conceptualization, investigation, methodology, software, data curation, writing – original draft, writing – review and editing, visualization, formal analysis, project administration.

## Conflicts of Interest

The authors declare no conflicts of interest.

## Supporting information




**Supporting File 1**: desc70115‐sup‐0001‐SuppMat.pdf

## Data Availability

The data that support the findings of this study are available from the authors upon reasonable request.
