# Supplementary material for: Explaining the Comprehension–Production Vocabulary Gap Through Neural Networks and Cross‐Syndrome Evidence: Insights From Williams Syndrome
Source: Dev Sci. 2026 Jan 8;29(2):e70115. doi: 10.1111/desc.70115 (PMC12781623; doi:10.1111/desc.70115)
Supplement: Supplementary file 1 — Supporting File 1: desc70115‐sup‐0001‐SuppMat.pdf [file DESC-29-e70115-s001.pdf]

Supporting Information

Explaining the Comprehension–Production Vocabulary Gap through Neural Networks and Cross-Syndrome Evidence: Insights from Williams Syndrome

Dean D'Souza[1], Hana D'Souza[1], Julien Mayor[2], Ángel Eugenio Tovar[3,4]

[1] Centre for Human Developmental Science, School of Psychology, Cardiff University, UK
[2] Department of Psychology, University of Oslo, Norway
[3] Facultad de Psicología, Universidad Nacional Autónoma de México, México
[4] Centro de Ciencias de la Complejidad, Universidad Nacional Autónoma de México, México

1. Computational Model Details

1.1 Model Architecture

The computational model consisted of two Self-Organising Maps (SOMs), one dedicated to processing auditory input and the other to visual input. These maps were fully interconnected via Hebbian learning, establishing potential all-to-all connections between neurons in the auditory and visual maps, with Hebbian weights initialised to small random values (< 0.5).

In the Reference Model, each SOM was implemented as a 12X12 grid of 144 neurons, where each neuron was associated with a weight vector (w_i) of the same dimensionality as the input vectors (x). The SOM weights were initially randomised within the range [0–1]. Neurons self-organised based on their proximity to the input vector, enabling topographic representation of the input space.

1.2 Processing and Learning Dynamics in the SOMs

When an input vector (x) is presented to a SOM, the Best Matching Unit (BMU) is the neuron whose weight vector (w_i) minimises the Euclidean distance:

$$BMU = \underset{i}{\operatorname{argmin}}\{\| x - w_i \|\} \quad (1)$$

The neighbourhood function controls the extent to which neurons surrounding the BMU adjust their weight vectors during learning. Neurons that are spatially closer to the BMU are updated more substantially, while more distant neurons undergo smaller adjustments. This mechanism promotes topographical organisation in the SOM, where similar inputs are represented by neighbouring units.

We used a Gaussian function (Equation 2), centred on the BMU, to determine the degree of weight adaptation for each neuron as a function of its *distance* from the BMU.

$$gnf = \exp\left(-\frac{distance^2}{2 \times \sigma_t^2}\right) \quad (2)$$

Over the course of training, the effective radius of the neighbourhood gradually shrinks, allowing the network to transition from broad activations to more fine-grained ones. The radius decay was implemented linearly, with the neighbourhood radius parameter sigma (σ) linearly decreasing from an initial value of $\sigma_{t=1}$ = 3 to a final value of $\sigma_{t=T}$ = 1 by the end of training epochs, following Equation 3.

$$\sigma_t = \sigma_{t=1} - (\sigma_{t=1} - \sigma_{t=T}) \times \frac{t-1}{T-1} \quad (3)$$

Where *t* is the current epoch and *T* is the total epochs (400 in our simulations, as the Reference Model stabilised at this value).

SOM weights are updated based on the discrepancy between the current input stimulus (x) and each neuron's internal representation (w), known as the *error*. This error is then scaled by two factors: the Gaussian neighbourhood function (gnf) and a learning rate (α) that decays over time. This time-dependent learning rate is a standard feature of the original SOM algorithm (Kohonen, 2013) and ensures proper self-organisation throughout training. The update rule is formalised as:

$$som_{t+1} = som_t + (\alpha_t \times gnf \times error) \quad (4)$$

α was initialised at $\alpha_{t=1} = 0.9$ and decreased linearly over time to $\alpha_{t=T} = 0.1$ according to the following Equation 5:

$$\alpha_t = \alpha_{t=1} - (\alpha_{t=1} - \alpha_{t=T}) \times \frac{t-1}{T-1} \quad (5)$$

1.3 Hebbian Learning

Hebbian connections between the auditory and visual SOMs were initialised with random values in the range [0–0.5]. Their update followed a standard Hebbian learning rule, in which the weight change was proportional to the activation levels of the connected units:

$$w_{(t+1)} = w_{(t)} + (1 - e^{-\beta * act_i^{visual} * act_j^{auditory}}) \quad (6)$$

Where w is the Hebbian connection between neuron *i* (visual) and neuron *j* (auditory), act_i^{visual} and $act_j^{auditory}$ are the activation levels of neurons *i* and *j* respectively, computed from the Gaussian neighborhood function described above. The BMU receives the highest activation (1) and activation values decrease with distance to the BMU. Beta (β) is the learning rate for the Hebbian weights and increased gradually over time according to: $\beta_{t+1} = \beta_t \times 1.0009$. This increasing approach has been used in previous models of lexical development (Althaus & Mareschal, 2013). Unlike the SOM parameters (the neighbourhood radius σ and learning rate α) which decay over time to support topographic organisation, the Hebbian learning rate increases, reflecting the idea that as map organisation becomes more reliable, the system more confidently establishes associations between maps.

To prevent divergence of Hebbian weights, all connections are normalised after each update:

$$w_{(t+1)} = \frac{w_{(t+1)}}{\max(W)} \quad (7)$$

Where *W* denotes the full Hebbian weight matrix.

1.4 Input Coding

The model's vocabulary comprised 20 word-object pairings, each representing a distinct category. Every category included a verbal label and a corresponding visual referent represented by 8 exemplars, yielding 160 unique input vectors per sensory modality.

The category exemplars were generated from 10-dimensional prototype vectors. Given the focus of this work on modelling the emergence of the comprehension–production gap through differences in auditory and visual statistical regularities, we applied a strict criterion that the only distinction between input modalities was the level of within-category variability. The same set of prototype vectors was used to generate both auditory and visual inputs, and for implementation simplicity these were constructed arbitrarily by assigning non-repeating values from 1 to 10 across the ten vector dimensions and rescaling them to the range [0.1, 1]. Gaussian noise was added to generate 8 exemplars per prototype vector, with greater noise applied to visual inputs (σ = 0.25) than to auditory inputs (σ = 0.05).

1.5 Model Performance Metrics

Comprehension and Production. Lexical development was tracked after each training epoch using comprehension and production tests. In comprehension, the 160 auditory inputs (20 categories x 8 exemplars) were presented, and the corresponding visual activation was assessed. Specifically, the auditory BMU for each input was retrieved. For each auditory BMU, the visual neuron with the strongest Hebbian connection was identified and compared to the expected category. A trial was considered correct if the closest stored category in the visual map matched the intended category presented during training (any of its 8 exemplars). A category was considered successfully learned if at least 80% of its exemplars were correctly classified.

Production tests followed the reverse procedure: the 160 visual inputs were presented, and for each visual BMU, the auditory neuron with the strongest Hebbian connection was identified and compared to the expected auditory label.

To quantify lexical development across training, we tracked the number of correctly processed words at each epoch, producing comprehension and production trajectories over 400 training epochs for each simulation. To summarise these trajectories, we computed the Area Under the Curve (AUC) using numerical integration (the trapezoidal rule). AUCs were calculated for each simulation run and then averaged across runs to obtain a summary measure for each model configuration. When plotted, these trajectories place training epochs on the horizontal axis and number of correct words on the vertical axis (see the first row of results in figures SI-1, SI-2, SI-3, SI-4 and SI-5 provided in this document). Thus, the AUC can be interpreted as an estimate of the total number of words successfully accessed throughout training.

Quantization Error. To assess the internal representational quality of the maps after each epoch, we calculated the mean Quantization Error (QE). For each input presented to the SOM a QE value was defined as:

$$QE = || x_i - w_{\_BMU} || \quad (7)$$

where x_i is the input vector, and $w_{\_BMU}$ is the weight vector of the BMU corresponding to x_i. Higher QE values indicate that the BMU's weight vector is further from the input in the feature space, reflecting a less faithful encoding of that stimulus. Conversely, lower QE values indicate that the BMU's weight vector is closer to the input vector, meaning the learned representation more closely matches (is more tightly aligned with) the stimulus' actual features.

Categorisation Metric. We analysed how objects were grouped across the map. For each exemplar we determined its BMU. We then counted how many different BMUs were activated by the entire set of exemplars within an epoch. This count was then divided

by the total number of neurons in the map (e.g., 144 in the 12×12 Reference Model) and normalised to range between 0 and 1.

In this way, a score close to 0 indicates prototype-based organisation, as it means that a small number of distinct BMUs were activated across all exemplars. This reflects high category compactness and abstraction. In contrast, scores close to 1 reflect exemplar-based organisation, where each exemplar tends to map to a distinct neuron, resulting in more dispersed representations.

1.6 Modeling Williams Syndrome

To explore variations in the model that lead to WS-like lexical profiles, we manipulated three components.

Map Size Reduction was implemented by using 9x9 maps (i.e., totaling 81 neurons in each map). During pilot simulations, we tested different map sizes (e.g., 11×11, 10×10, 9×9) and confirmed that progressively reducing the number of neurons was associated with increasingly delayed performance. We stopped at 9×9 to avoid artificially forcing excessive category compactness due to overly constrained representational space.

Input Noise was modeled through Gaussian noise, which was added to input vectors during training. For each exemplar, noise (with zero mean and σ = 0.15) was selectively applied to a subset of the input features (i.e., 4 out of 10 dimensions). The affected dimensions varied across model runs but remained fixed within each run. This method introduces structured variability across exemplars and results in learning delays within the model. In contrast, other forms of noise, such as noise applied uniformly across all input dimensions, produced a bifurcated effect, either having little to no impact on the model's performance or severely disrupting performance, thus limiting their usefulness for modeling atypical learning.

Neighbourhood Function Disruption involved altering the radius and decay of the neighbourhood in the SOMs. In the Reference Model, the radius sigma decreased linearly from 3 to 1 across training epochs, supporting gradual refinement of the map. In contrast, for the WS model, we used a fixed radius sigma of 0.25 throughout training. This value sharply limited the spread of the Gaussian neighbourhood function, reducing local collaboration among neighbouring neurons and weakening the map's capacity to form well-organised categories.

2. Simulation Results

The following figures (SI-1, SI-2, SI-3, SI-4, SI-5), provide a summary of the simulation results for each tested model, with the Reference Model consistently displayed in the leftmost column for comparison.

The first row presents mean vocabulary comprehension and production trajectories, with standard deviation shown in shaded areas. The area under the curve (AUC) is reported, with bold values indicating significant delays relative to the reference model (see statistical comparisons in Table SI-T1).

The second row presents scatter plots illustrating the relationship between comprehension and production for each model, generated by randomly selecting five data points per model run (for a total of 100 points across the 20 runs). A shape-constrained additive model (SCAM) with a monotonic positive increase constraint and *m_basis* = 3 was applied for smoothing, shown in red, with the 95% CI represented by the shaded area. The same SCAM fit for the reference model (in black) is included for direct comparison. Models with a reduced comprehension-production gap exhibit SCAM fits that pass above the reference model curve, whereas those with an increased gap fall below it.

The third and fourth rows display Quantization Error (QE) trajectories for auditory (third row) and visual (fourth row) maps, with 20 simulation runs overlaid in colour. The reference model (grey) trajectories are included for comparison. Some models exhibit consistently higher QE values with a slower decline (e.g., see models with map size reductions Fig SI-1), indicating delayed learning trajectories. Other models show flatter QE trajectories, with initially lower values than the reference model but with substantial fluctuations (e.g., see models with disruptions in neighborhood functions Fig SI-3), which suggest that BMU selection occurs independently of surrounding neuron values, leading to rapid but highly localised weight adjustments, indicative of exemplar-based organisation.

The fifth and sixth rows illustrate the final category organisation within the SOMs for one run of each model. Each colour represents a distinct category, with its position indicating the BMUs assigned to that category. Below each figure, the model's mean categorisation score and standard deviation are reported, where values near 1 indicate more exemplar-based processing and values near 0 reflect prototype-based processing. Some models (e.g., Reference Model) exhibit well-clustered categories, while others (e.g., the WS visual map, Fig SI-4) show a dispersed, collage-like pattern, reflecting exemplar-based processing.

Notably, the collage-like SOMs align with models exhibiting atypical QE patterns, reinforcing that QE and SOM structure both reflect difficulties in category organisation. Furthermore, these models also show the most atypical comprehension-production gaps, confirming a strong link between disrupted category formation and lexical development irregularities.

2.1 Effects of Map Size Reductions

Reducing the number of neurons in each SOM led to lexical delays (Fig SI-1). When both the auditory and visual maps were reduced (column 2), delays were observed across lexical development. A reduction in the visual map selectively impaired visual QE and visual categorisation, while auditory QE and categorisation were preserved. Conversely, reducing the auditory map affected auditory QE and categorisation without altering visual QE and categorisation. Since lexical development relies on cross-modal mappings, both types of reductions ultimately contributed to delays in vocabulary acquisition. However, map size reductions did not affect the asymmetry between comprehension and production, as seen in the second row, where the red SCAM fits in the tested models run parallel to those of the reference model.

Figure SI-1. Effects of Map Size Reductions

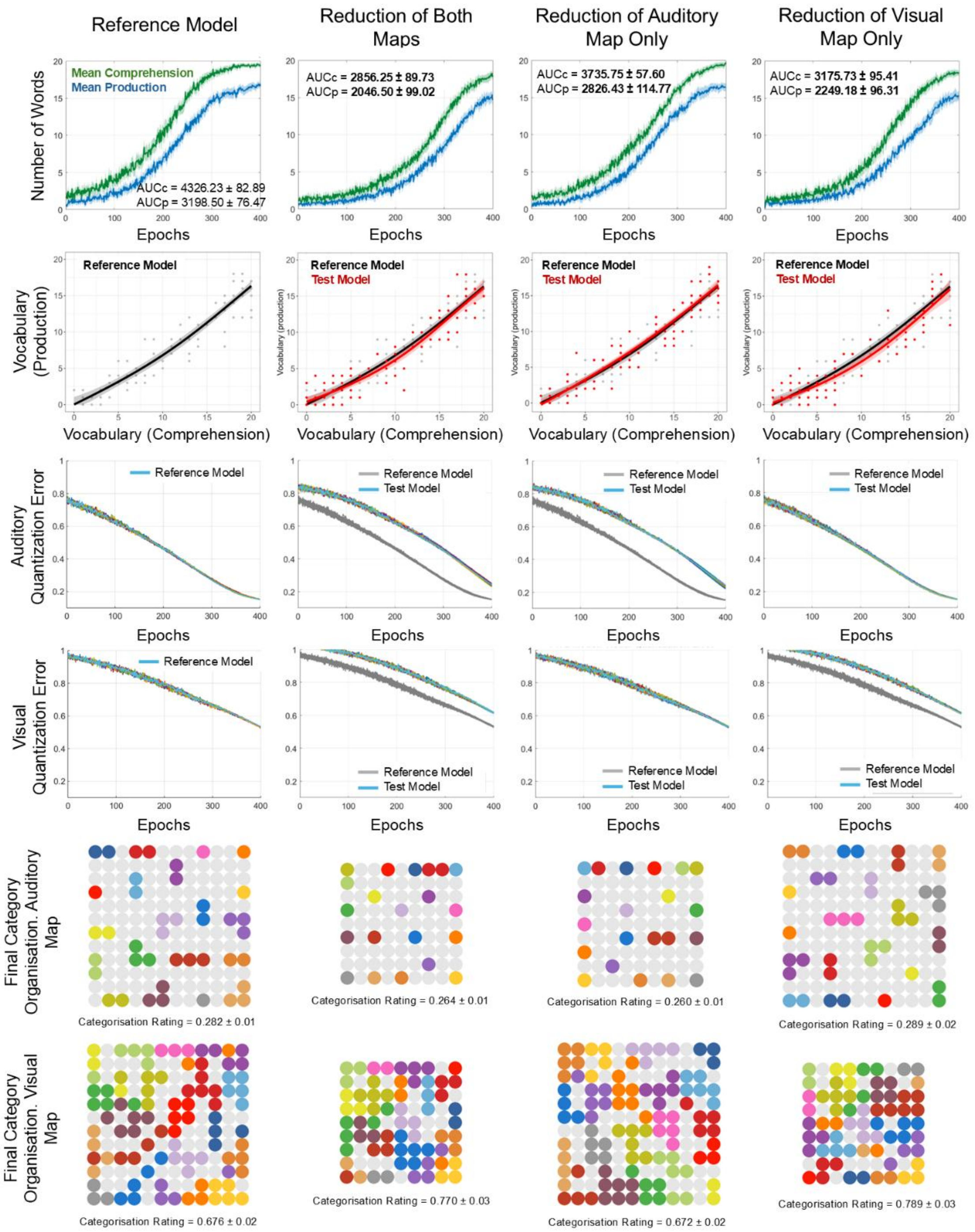

2.2 Effects of Input Noise

Adding noise to the inputs also led to delays in lexical development (Fig. SI-2). The results show a similar pattern to Figure SI-1, because adding visual input noise impaired visual QE and categorisation while leaving auditory QE and categorisation unaffected, and vice versa. Lexical acquisition was delayed, as reflected in both comprehension and production trajectories (first row). However, input noise did not affect the comprehension-production gap, as shown in the second row, where the red SCAM fits remain parallel to those of the reference model.

Figure SI-2. Effects of Input Noise

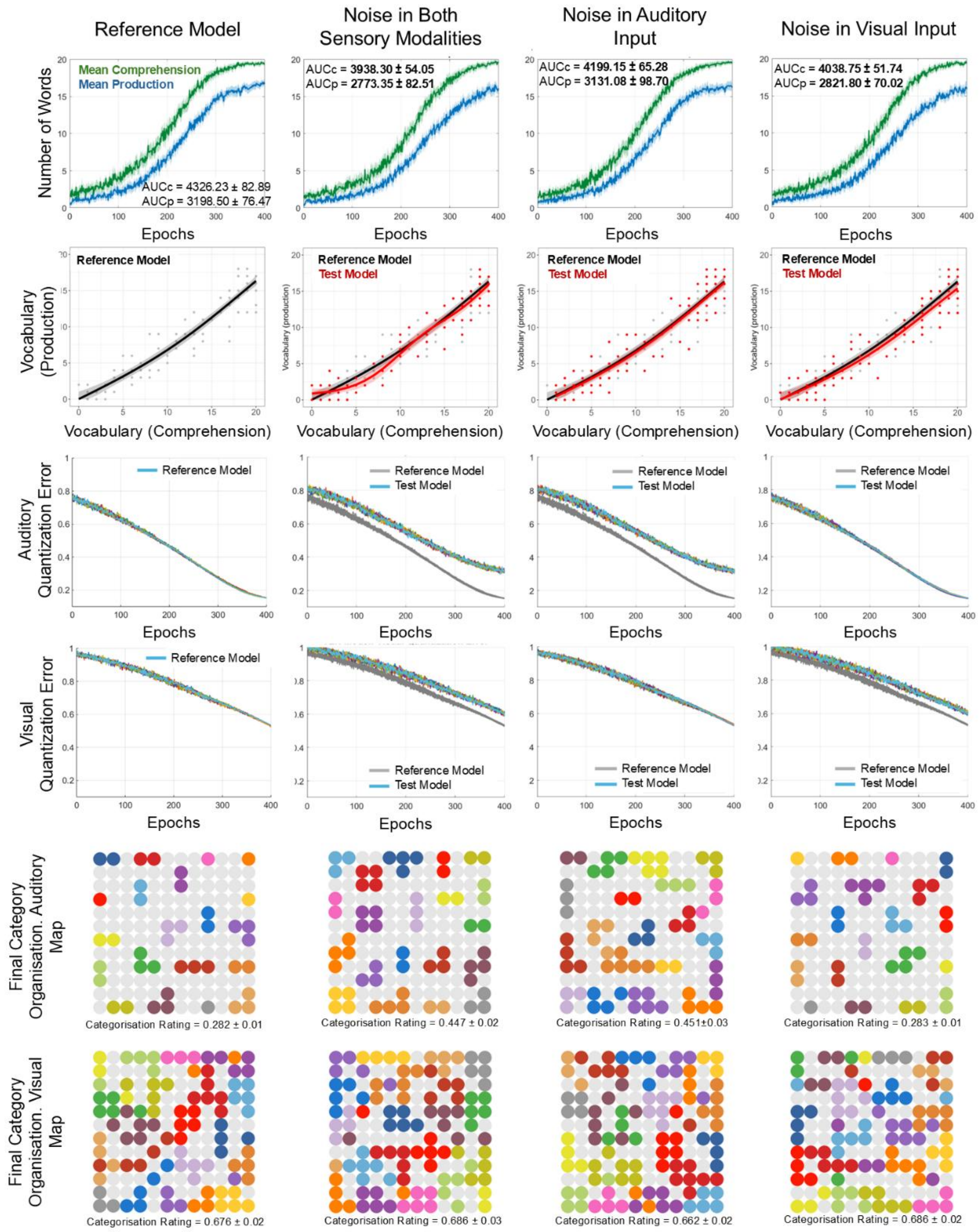

2.3 Disruptions in Neighborhood Function

Changes in the neighborhood function had pronounced but modality-specific effects (SI-3). Disrupting both maps led to an abrupt and exaggerated lexical acquisition, alongside atypical QE patterns with rapidly decreasing values. However, categorisation was differentially affected: while the visual modality shifted toward exemplar-based organisation, the auditory modality preserved prototype-based structure. Given that auditory categories exhibit lower variability than visual ones, this suggests that the interaction between input statistics and neighborhood disruptions impacts visual categorisation more severely. Notably, an auditory-only lesion (last column) resulted in a delayed trajectory but with comprehension showing greater growth than production. In contrast, a visual-only lesion inverted the comprehension-production gap, with production showing a relative advantage—aligning with a pattern sought for WS; however, this disruption alone does not capture the characteristic vocabulary delays.

Fig SI-3 Disruptions in Neighborhood Function

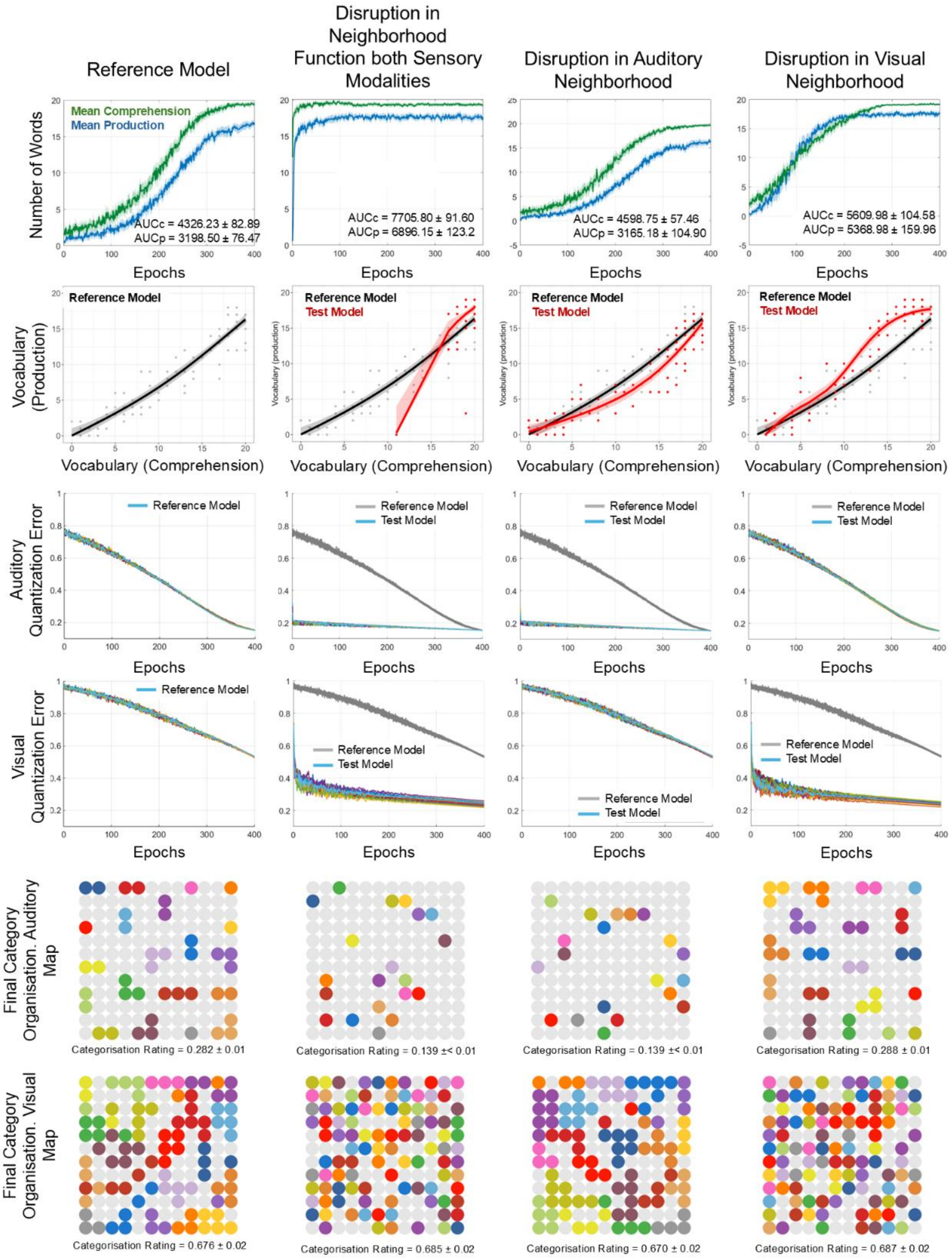

2.4 Effects of Combined Manipulations

We found that map size reductions and input noise led to lexical delays, while disruptions to the neighborhood function altered the comprehension –production gap. To examine how these effects interact, we tested combinations of lesions to identify a pattern that best matched WS. The last column shows that the combination of reduced maps in both modalities, noise in both modalities, and a visual neighborhood disruption successfully captures the key WS features: delayed acquisition alongside a reduced comprehension-production gap. Notably, this combination also disrupts visual categorisation, with atypical visual QE, while auditory categorisation remains preserved, though auditory QE indicates slower learning compared to the reference model. Other combinations capture partial aspects of WS but fail to fully meet the two key criteria.

Fig SI-4 Effects of Combined Manipulations

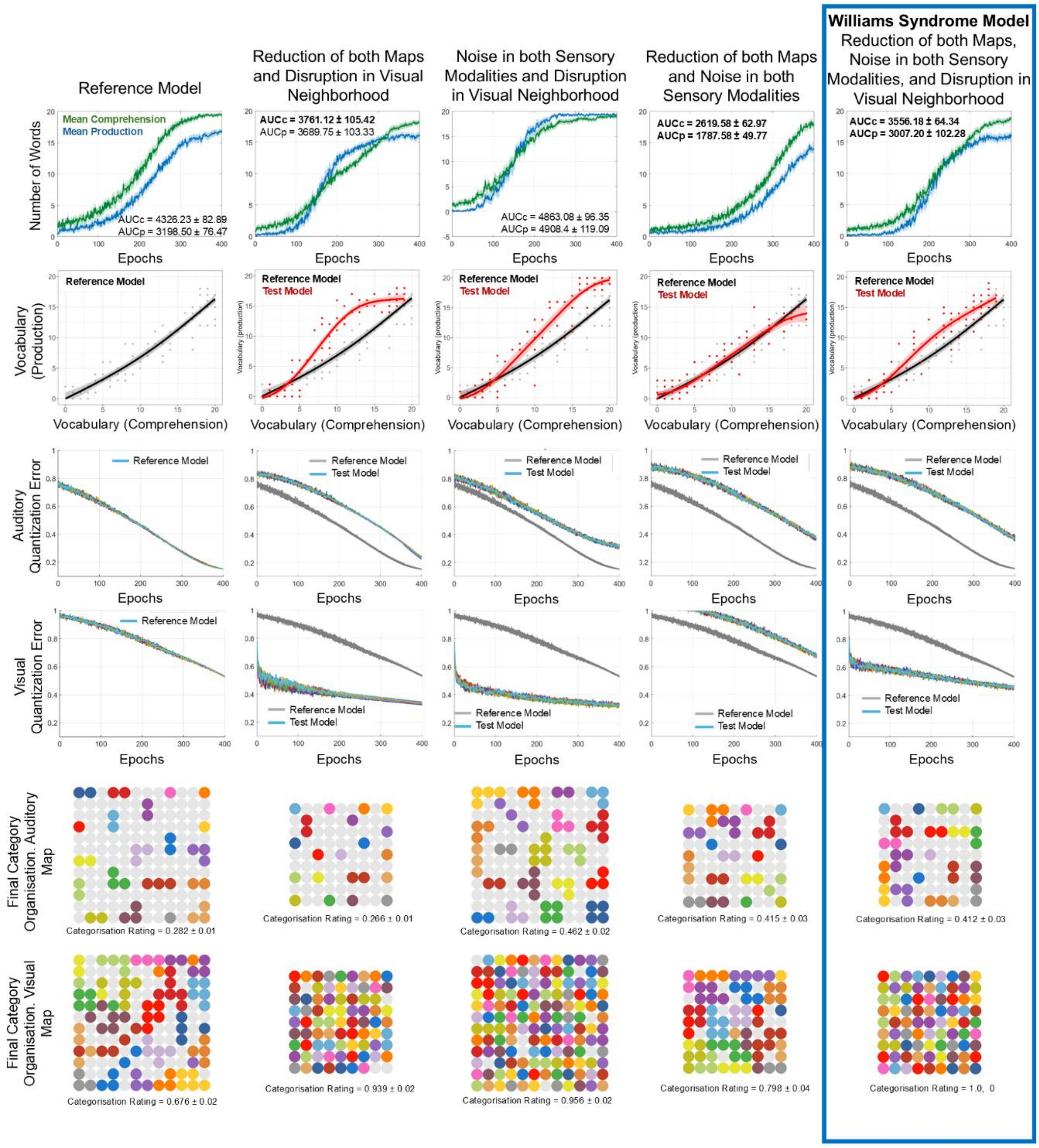

2.5 Exploring WS Patterns through Visual Disruptions

Finally, to investigate whether the WS pattern could primarily emerge from visual-specific disruptions, we tested models with alterations restricted to the visual modality (columns 2, 4, and 5) and one combining a visual and an auditory disruption (column 3). While all these models exhibited a reduced comprehension-production gap due to a relative advantage in production (attributed to disruption in the visual neighborhood), none successfully captured the characteristic lexical delays of WS. These results suggest that incorporating domain-general disruptions, such as map size reductions and input noise across both modalities, is essential to fully capture the WS pattern.

Fig SI-5 Exploring WS Patterns through Visual Disruptions

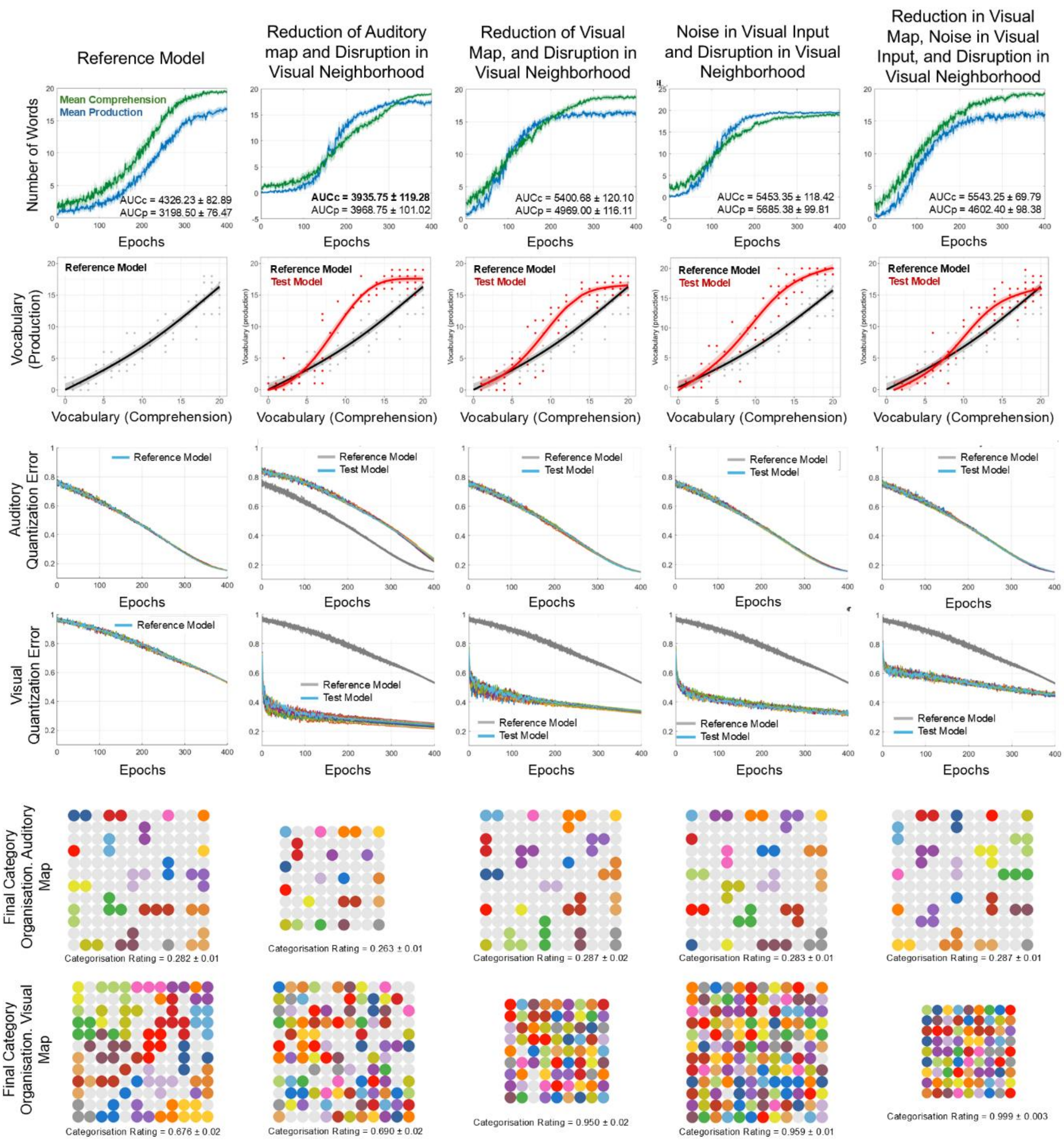

2.6 Statistical Analysis

In Table SI-T1 we present comprehension and production deltas, calculated from AUC measures, where negative values indicate delays relative to the Reference Model (centered at 0). Models showing significant delays (based on one-tailed independent-samples t-tests, FDR-corrected) are highlighted in blue. For models with both comprehension and production delays we conducted a binomial sign test, mirroring the analysis used for empirical data, to assess whether lexical production values were significantly above the Reference Model's SCAM prediction. Only one model (WS Model) showed a significant result, with 62 data points above the reference model (p = 0.01, 95% CI 0 [0.53, 1]).

Table SI-T1

Lexical Delays and Comprehension–Production Gap Analysis Across Tested Models

Model	Lexical Delay Analysis				Comp-Prod Gap Analysis (Binomial Sign Test)	
	Comprehension		Production			
	ΔComp	*t(p, FDR)*	ΔProd	*t(p, FDR)*	% of Production data Points above Reference Model Fit	*p*
**Reference Model**	0.00		0.00			
RMap aud&vis	-1469.98	-53.81 (p < .001)	-1152.00	-41.18 (p < .001)	45	0.86
RMap vis	-1150.50	-40.71 (p < .001)	-949.33	-34.52 (p < .001)	36	1.00
RMap aud	-590.48	-26.16 (p < .001)	-372.08	-12.07 (p < .001)	44	0.90
IN aud&vis	-387.93	-17.53 (p < .001)	-425.15	-16.9 (p < .001)	35	1
IN vis	-287.48	-13.16 (p < .001)	-376.70	-16.25 (p < .001)	37	1
IN aud	-127.08	-5.39 (p < .001)	-67.43	-2.42 (p = 0.022)	41	0.97
DNF aud&vis	3379.58	122.35 (p = 1.0)	3697.65	113.69 (p = 1.0)		
DNF vis	1283.75	43.02 (p = 1.0)	2170.48	54.75 (p = 1.0)		
DNF aud	272.53	12.08 (p = 1.0)	-33.32	-1.15 (p = 0.244)		
RMap aud&vis; DNF vis	-565.00	-18.84 (p < .001)	491.25	17.09 (p = 1.0)		
IN aud&vis; DNF vis	536.85	18.89 (p = 1.0)	1709.90	54.03 (p = 1.0)		
RMap aud&vis; IN aud&vis	-1706.65	-73.32 (p < .001)	-1410.93	-69.16 (p < .001)	46	0.82
**WS Model** RMap aud&vis; IN aud&vis; DNF vis	**-770.05**	-32.82 (p < .001)	**-191.30**	-6.7 (p < .001)	**62**	**0.01**
RMap vis; DNF vis	1074.45	32.93 (p = 1.0)	1770.50	56.95 (p = 1.0)		
RMap aud; DNF vis	-390.48	-12.02 (p < .001)	770.25	27.19 (p = 1.0)		
IN vis; DNF vis	1127.13	34.87 (p = 1.0)	2486.88	88.45 (p = 1.0)		
RMap vis; NI vis; DNF vis	1217.03	50.23 (p = 1.0)	1403.90	50.39 (p = 1.0)		

Note: Model codes: RMap = reduced maps; IN = input noise; DNF = disrupted neighbourhood function; vis = visual; aud = auditory. p-values were corrected for multiple comparisons using False Discovery Rate (FDR), Bonferroni correction confirmed the same pattern of results.

References

Kohonen, T. (2013). Essentials of the self-organizing map. Neural Networks: The Official Journal of the International Neural Network Society, 37, 52-65. https://doi.org/10.1016/j.neunet.2012.09.01
